# Strigolactone analogues induce apoptosis through activation of p38 and the stress response pathway in cancer cell lines and in conditionally reprogramed primary prostate cancer cells.

**DOI:** 10.18632/oncotarget.1849

**Published:** 2014-04-02

**Authors:** Claire B Pollock, Sara McDonough, Victor S. Wang, Hyojung Lee, Lymor Ringer, Xin Li, Cristina Prandi, Richard J. Lee, Adam S. Feldman, Hinanit Koltai, Yoram Kapulnik, Olga C Rodriguez, Richard Schlegel, Christopher Albanese, Ronit I. Yarden

**Affiliations:** ^1^ Department of Human Science, Georgetown University Medical Center, NW Washington DC; ^2^ Lombardi Comprehensive Cancer Center, Georgetown University Medical Center, NW Washington DC; ^3^ Department of Pathology, Georgetown University Medical Center, NW Washington DC; ^4^ Center for Cellular Reprogramming, Georgetown University Medical Center, NW Washington DC; ^5^ Department of Biostatistics, Bioinformatics and Biomathematics Georgetown University Medical Center, NW Washington DC; ^6^ Department of Chemistry, University Turin, Torino, Italy,; ^7^ Massachusetts General Hospital, Boston, MA,; ^8^ Department of Ornamental Horticulture, Agricultural Research Organization (ARO), Bet Dagan, Israel; ^9^ Department of Field Crops and Natural Resources Institute of Plant Sciences, Agricultural Research Organization (ARO), Bet Dagan, Israel

**Keywords:** Plant hormone, cell cycle arrest, apoptosis, stress response, p38-MAPK

## Abstract

Strigolactones are a novel class of plant hormones produced in roots and regulate shoot and root development. We have previously shown that synthetic strigolactone analogues potently inhibit growth of breast cancer cells and breast cancer stem cells. Here we show that strigolactone analogues inhibit the growth and survival of an array of cancer-derived cell lines representing solid and non-solid cancer cells including: prostate, colon, lung, melanoma, osteosarcoma and leukemic cell lines, while normal cells were minimally affected. Treatment of cancer cells with strigolactone analogues was hallmarked by activation of the stress-related MAPKs: p38 and JNK and induction of stress-related genes; cell cycle arrest and apoptosis evident by increased percentages of cells in the sub-G1 fraction and Annexin V staining. In addition, we tested the response of patient-matched conditionally reprogrammed primary prostate normal and cancer cells. The tumor cells exhibited significantly higher sensitivity to the two most potent SL analogues with increased apoptosis confirmed by PARP1 cleavage compared to their normal counterpart cells. Thus, Strigolactone analogues are promising candidates for anticancer therapy by their ability to specifically induce cell cycle arrest, cellular stress and apoptosis in tumor cells with minimal effects on growth and survival of normal cells.

## INTRODUCTION

The strigolactones (SLs) are members of a class of plant hormones that control shoot branching architecture by inhibiting growth and self renewal of axillary meristem cells [1, 2]. We have recently shown that strigolactone analogues induce growth arrest and apoptosis in breast cancer cell lines [3], thereby adding the SLs to the list of plant-derived effective anti-cancer agents that are being assessed for their ability to inhibit the growth and survival of human cancer cell lines [4-6] [7, 8] [9, 10].

The p38 MAPK is a member of the conserved MAPK family of dual serine-theronine kinases that respond to variety of internal and external stimuli. The p38-MAPK pathway is comprised of four genes; α, β, γ, δ with p38α being the most abundant isoform [11]. p38α responds to many stress stimuli including reactive oxygen species, UV irradiation, hypoxia, and pro- inflammatory cytokines. Considerable data imply that P38α functions as a tumor suppressor, inducing cell cycle arrest, differentiation and apoptosis [11, 12]. Mutations in (p38α) were identified by massive parallel sequencing in human tumors [11, 13] and xenografts of cells deficient in p38α signaling exhibited higher tumor burden than their wild type counterparts [14-17]. Furthermore, p38 was found to be a marker for tumor dormancy and as an inhibitor of self-renewal [18]. Conversely, recent reports have suggested that p38α is involved in cell survival and increased invasion in prostate cancer cells and other advanced tumor types [19-21], suggesting an addiction to p38 MAPK activity that was correlated with poor prognosis [22]. Thus, the role and function of p38 in tumorigenesis is complex and may be tissue and context dependent.

The Jun N-terminal Kinase (JNK) pathway also plays a major role in cellular stress response. JNK1 and JNK2 are the most abundantly expressed isoforms of the JNK family. JNK1/2 exert their effects via regulation of multiple target genes that contain binding sites for the transcription factor, AP1 (JUN-FOS)[11, 23]. As with p38, JNK1/2 have been described as either negative or positive regulators of cell growth and have been associated with both pro-apoptotic and pro-survival activities. Recent studies have found that JNK activation enhanced chemotherapy-induced apoptosis in multiple cancer cells [11], while in prostate cancer, especially in tumors that harbor mutations in PTEN, activated JNK1/2 supports proliferation and survival [23, 24]. Collectively it appears that the type of response depends on intensity and duration of the signals as well as cell type.

Mechanistically, the JNK and p38 MAPK pathways share several upstream regulators and there is evidence of cooperation and synergy between downstream targets such as HSP27, HSP70 and FOXO4 [25, 26]. Perhaps one reason for overexpression and constitutive activation of the stressed-related pathways in prostate and other types of advanced tumors is the constant insults present in the cancer cell per se including enhanced DNA damage and accumulation of reactive oxygen species [27].

Our previous work had found that the growth arrest and apoptosis induced by SL analogues in breast cancer cell lines occur in part through the activation of the stress activated MAPKs, p38 and JNK1/2 and reductions in ERK1/2 and AKT activity. Here we further define the mechanisms of action, as well as the overall applicability of SLs as cancer therapeutics. We show that strigolactone analogues changes the genomic landscape of cells and despite cells addiction to stress signaling, SL are capable of eliciting a significant stress response including induction of multiple heat shock proteins, phosphorylation of p38MAPK and JNK1/2 and induction of their targets such as HSP27, HSP70 and FOXO4 [25, 26]. In addition, we employed the new cell culture technology of conditionally reprogrammed primary cells (CRCs) that were established from patient-matched prostate normal and tumor tissues. The reprogrammed cells can grow indefinitely in the presence of irradiated feeder cells and ROCK inhibitor and can model individual patient responses [28, 29].

Prostate tumor CRCs, are significantly more sensitive to SLs than their patient-matched normal CRCs. Together, we show that SL analogues produce their cytotoxic effect in part through activating p38 MAPK and a prolonged stress response in both transformed cell lines and in patient-derived CRCs.

## RESULTS

### Strigolactone analogues are effective growth inhibitors of a diverse range of cancer derived cell lines

**Figure 1:**
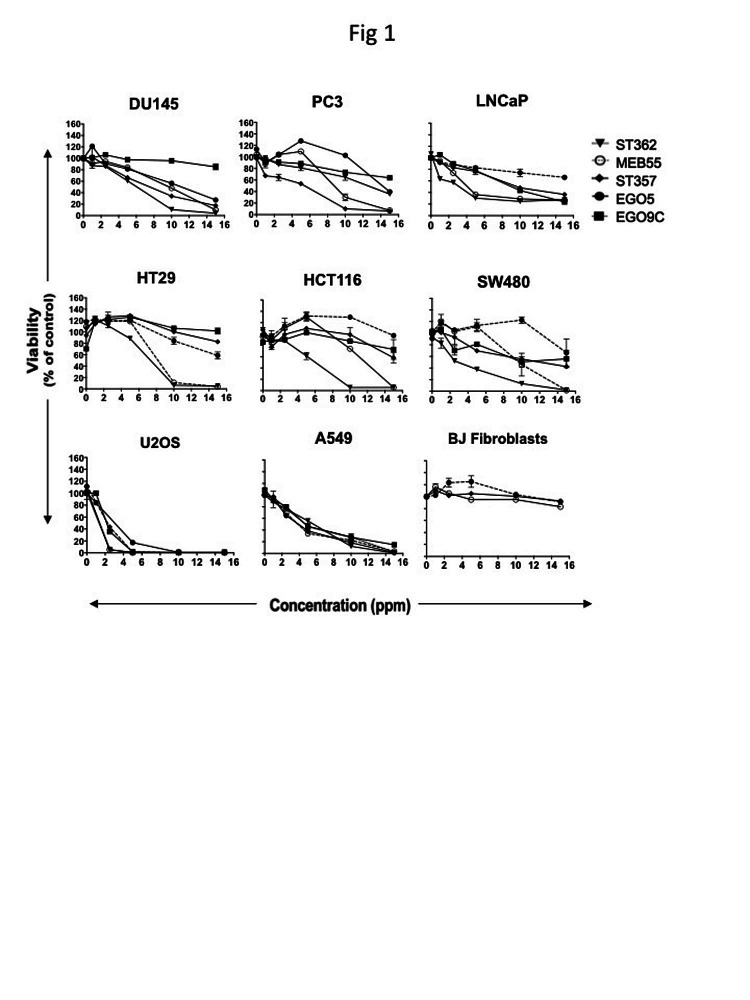
Strigolactone analogues induce cell death in human cancer cell lines. A. Cancer cell lines from the prostate; LNCaP, DU145 and PC3, Colon; HT29, HCT116 SW480, Osteosarcoma; U2OS and Lung; A549, as well as the normal BJ fibroblast cell line were seeded into 96 well plates in normal growing media. The following day media was replaced with phenol red-free DMEM supplemented with 10% charcoal stripped serum and the indicated doses of Strigolactone analogues (four replicates for each treatment group or vehicle control). Cell viability was assayed by XTT after 3 days. Graphs are representative of the mean ± standard deviations (SD) of two independent experiments.

To determine whether synthetic SL analogues inhibit the proliferation of cell lines derived from different types of cancers, a diverse cohort of human cancer lines was collected comprising prostate, colon, lung, osteosarcoma, and leukemia derived lines. Cancer cell lines and BJ normal fibroblast cells were treated with each SL analogue at a dose range of 1 to 20 ppm (equivalent to 2-60 μM; Figure 1, Figure S1A and Table 1). After 3 days, XTT cell proliferation assays were carried out and the changes in proliferation and cell survival and are presented as a percent of vehicle controls. All five SL analogues (EG5, EG9c, ST357, ST362 and MEB55) inhibited cancer cell growth albeit to varying degrees. In general, ST362 and MEB55 were the most potent inhibitors with the concentrations required to achieve 50% inhibition of cell viability (IC_50_) at day 3 of treatment ranging from 2.8 ppm to 12.8 ppm. EG5, EG9c and ST357 were less potent inhibitors of prostate, colon and lung cancer cell growth with IC_50_ values are mostly above 15 ppm. Interestingly the Osteosarcoma-derived line, U2OS exhibited a similar sensitivity to all SL analogues tested (IC_50_ = 2.7 to 4.5 ppm). None of the analogues tested-reached IC_50_ values in concentrations of up to 20 ppm in non-transformed BJ fibroblast cells (Figure 1). Table 1 summarizes the IC_50_ values for each cell line.

**Table 1:** The inhibitory concentrations (IC50) of Strigolactone analogues for cancer cells. Mean values are derived from 4 replicates of at least two independent experiments. The conversion between PPM and molarity is given for each analogue.

Tumor Cell Lines	IC50 (ppm) at 72 h				
	EG5 1ppm= 2.3 μM	EG9C 1ppm=3.2 μM	ST357 1 ppm=2.5 μM	ST362 1 ppm=2.0 μM	MEB55 1 ppm = 3.1 μM
Prostate PC3 DU145 LNCaP	>15 13 >15	>15 >15 11.5	5.0 8.5 11.4	10.3 7.5 2.5	8.8 10.8 2.99
Colon HT-29 HCT116 SW480	>15 >15 >15	>15 >15 >15	>15 >15 >15	7.3 5.2 2.9	8.2 10.4 9.7
Lung A549	4.7	15.5	4.9	5.2	5.1
Osteosarcoma U20S	3.9	4.5	4.5	2.8	2.7
Non-tumor cells BJ Fibroblasts	ND	>20	>20	ND	>20

### Strigolactone analogues inhibit cyclin B expression and induce a G2/M cell cycle arrest

**Figure 2:**
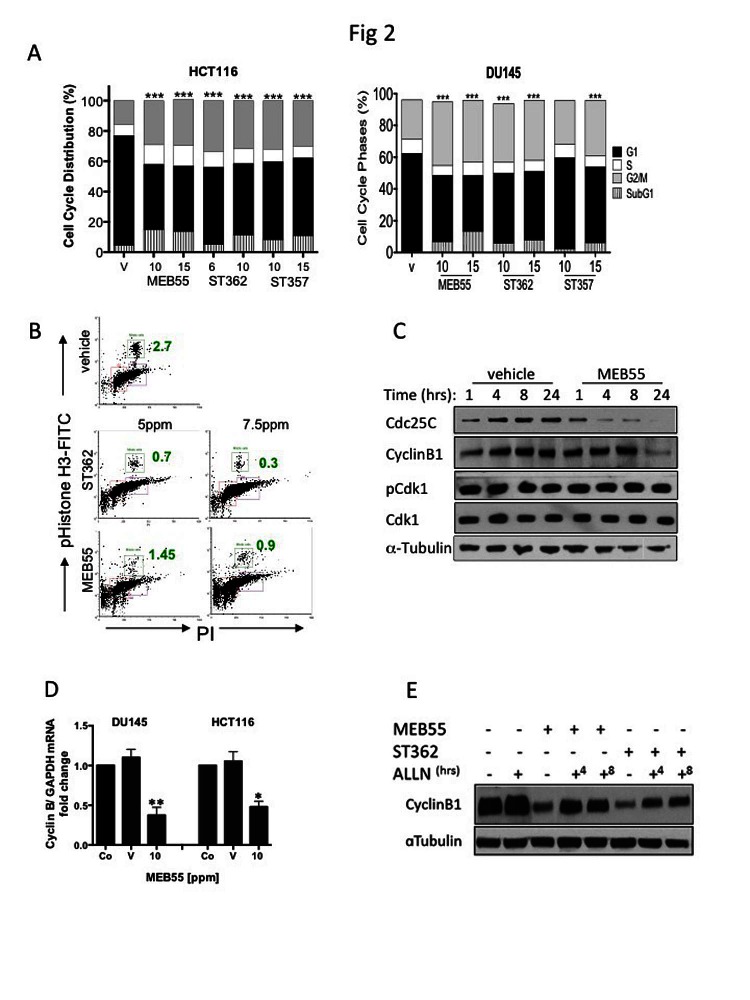
Effects of SL analogues on cell cycle regulation. A, The cell cycle distribution of HCT116 colon cancer and DU145 prostate cancer cells treated with the indicated doses of strigolactone analogues for 48 hr was analyzed by flow cytometry. Bar graphs represent mean ±SD of at least two experiments. *** P<0.001 as analyzed by Student *t-*test. B. The percent of treated HCT116 cells in M phase (green boxes and numbers) as assessed by FACS analysis of phospho-Ser10 Histone-H3 (vertical) versus DNA content (horizontal) is shown. The cells were treated with 5.0 or 7.5 ppm of either ST357 (middle panels or MEB55 (lower panels) and harvested after 48 hr. Immunoblot analyses of cell cycle regulatory proteins. DU145 cells were treated with 10 ppm of MEB or vehicle alone for the indicated times and the levels of cyclin B, Cdc25C, Cdk1 and Thr14-Cdk1 were determined. D. qRT-PCR for cyclin B1 mRNA expression in DU145 and HCT116 cells treated with vehicle or 10 ppm of MEB55. The experiment was repeated twice and graphs represent mean ± SD of triplicate wells from each experiment is shown vs. controls. * P<0.05, ** P<0.01 as analyzed by Student *t-*test. E. Cyclin B1 protein levels following proteasomal inhibition in DU145 cells treated with MEB55 or ST362. The cells were exposed to SLs for 24 hr prior and then treated with 10 mM ALLN for additional 4 (+^4^) or 8 (+^8^) hrs.

To assess the effect of SL treatment on the cell cycle, HCT116 (colon) and DU145 (prostate) cancer cells were treated with the SL analogues ST357, ST362 and MEB55 (chosen for their lower IC_50_ doses, see Table 1) for 48 hrs and cell cycle distribution was analyzed by flow cytometry following propidium iodide (PI) staining (Figure 2A). SL treatments induced significant increases in the proportion of cells in the G2/M phase and a concomitant decrease in the G1 fraction (p<0.0001) in all conditions studied with the exception of DU145 cells treated with 10 ppm of ST357 (p≤0.28). In addition, increased percentages of cells in the sub-G1 fraction were observed, indicating increased apoptosis (Figure 2A) (p< 0.0001). SLs treatment had similar effect on additional cancer lines (Figure S2).

Histone H3 phosphorylation on serine 10 (H3pS10) was used as a marker to distinguish between G2 and M arrest following SL treatment [30]. A reduction in the mitotic fraction in HCT116 cells from 2.7% in untreated cells to 0.9% and 0.3% in cells treated with 7.5 ppm ST362 and MEB55 respectively indicate that cells arrest at the G2 rather than the M fraction of the cell cycle (Figure 2B).

Cell cycle progression from G2 to mitosis is dependent on the timely accumulation and activation of the Cyclin B1/ Cdk1 complex [31]. Dephosphorylation of Cdk1 at threonine 14 is a critical step for the complex activation. Cyclin B1 levels are significantly decreased following 24 hrs of SL treatment in DU145 (Figure 2C) and HCT116 (Figure 2D) and so is the expression level of Cdc25C, the phosphatase responsible for Cdk1 dephosphorylation and activation (Figure 2C). However, no changes in protein levels or Cdk1 phosphorylation status at Thr14 were noted.

Cyclin B1 levels are regulated by several mechanisms including transcription and post-translational modifications that alter its stability. Quantitative Real-time PCR (qRT-PCR) revealed a two-fold or more decrease in cyclin B1 mRNA in HCT116 and DU145 respectively, following treatment with 10 ppm of MEB55 compared to vehicle controls (Figure 2D). We also found that SL analogues regulated cyclin B1 protein stability. Proteasome inhibition with ALLN for 4 or 8 hrs following 24 hrs exposure of DU145 cells to MEB55 or ST362 induced a partial rescue (ST362; 2 fold, MEB55; 1.3 fold) of cyclin B1 protein levels (Figure 2F). Taken together, SL analogues regulate cyclin B1 in part by reduced mRNA synthesis and in part by enhanced protein degradation and employ alternative mechanisms to induce G2 arrest.

### SL treatment induces a non-reversible decrease in cell viability

**Figure 3:**
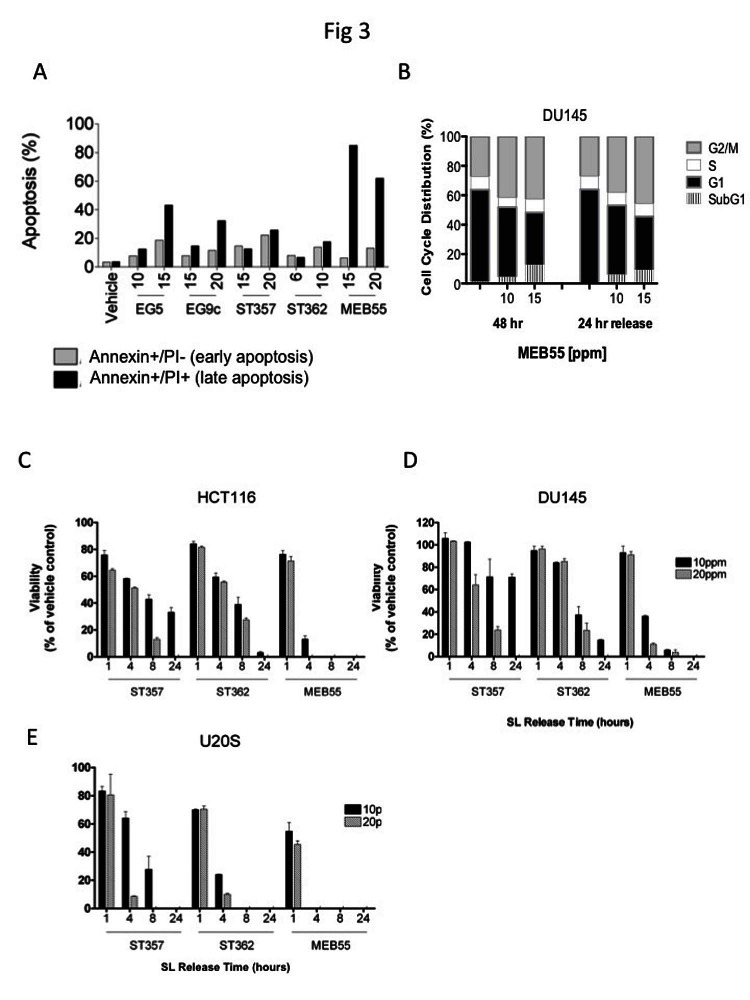
Strigolactone analogues induce cell death via apoptosis. A, HCT116 cells were treated with the indicated concentrations of SLs for 24 hr. Cells were co-stained with annexin-V and PI and analyzed by flow cytometry. The distribution of HCT116 cells in early (Annexin-/PI+, *gray bars*) and late (Annexin+/PI+, *black bars*) apoptosis following SL treatment. B. Cell cycle distribution of DU145 cells treated with the indicated doses of MEB55 for 48 hr, washed with PBS and overlaid with fresh media without SL for additional 24 hrs and analyzed by flow cytometry. C-E. Assay of SL reversibility in cells. HCT116 (C), DU145 (D) and U2OS (E) cells were treated with the indicated SLs for the indicated times, the cells were washed with PBS and overlaid with fresh DMEM containing 10% FBS minus SLs. Changes in cell viability were assessed by an XTT assay and are shown as mean ±SD of triplicate wells and expressed as percent of control.

Since SL prolonged the G2/M phase of the cell cycle and increased the accumulation of cells in the subG1 fraction (Figure 2A and 2B), dual staining with Annexin V, and PI was employed. Annexin V is a marker of early apoptosis, while PI only stains the chromatin of cells with compromised plasma membrane and therefore is a marker of late apoptosis. In HCT116 cells all five SL analogues increased the percentage of early (Annexin V+/PI-) and late (Annexin V+/PI+) apoptotic cells in a dose dependent manner (Figure 3A) and similar results were observed in two additional colon cancer cell lines, (SW480, HT29, Figure S2).

Interestingly, we found that the inhibitory and apoptotic effects of SL analogues are non-reversible, as DU145 cells treated with MEB55 for 48 hrs were not able to recover and re-enter the cell cycle following removal of the analogue and replacement of the growth media with fresh growth media without MEB55 for additional 24 hrs (24 hrs release). Cell cycle analysis showed that similar proportions of cells remained arrested at the G2/M checkpoint and there was no appreciable change in the subG1 fractions (Figure 3B).

The rapid non-reversible effect of ST357, ST362 or MEB55 on cell viability of HCT116, DU145 and U2OS cells was determined following exposure of cells for 1, 4, 8 or 24 hrs after which the different SLs were removed and media was replaced with fresh growth media for additional 48 hrs release. While a short 1 hr exposure to any of the SL analogues produced only a modest non-reversible decrease in cell viability in the three cell lines (Figure 3C-E), 4hrs exposure was sufficient to cause significant non-reversible reduction in cell viability as measured by XTT assays. U2OS cells exhibited the greatest sensitivity (Figure 3E) to SLs treatment, which correlates with the lower IC_50_ values in this cell line (Figure 1, Table 1). MEB55 was the most potent SL, and by 4 hrs treatments at the concentration of 10 ppm, only a small fraction of the cells remained viable (HCT116 18%; DU145 39% and U2OS 0% cells). As expected a further decrease in cell viability was observed when the three cell lines were exposed to SL for 8 hrs and 24 hrs.

### Strigolactone analogues induce stress response signaling and inhibit survival signaling

**Figure 4:**
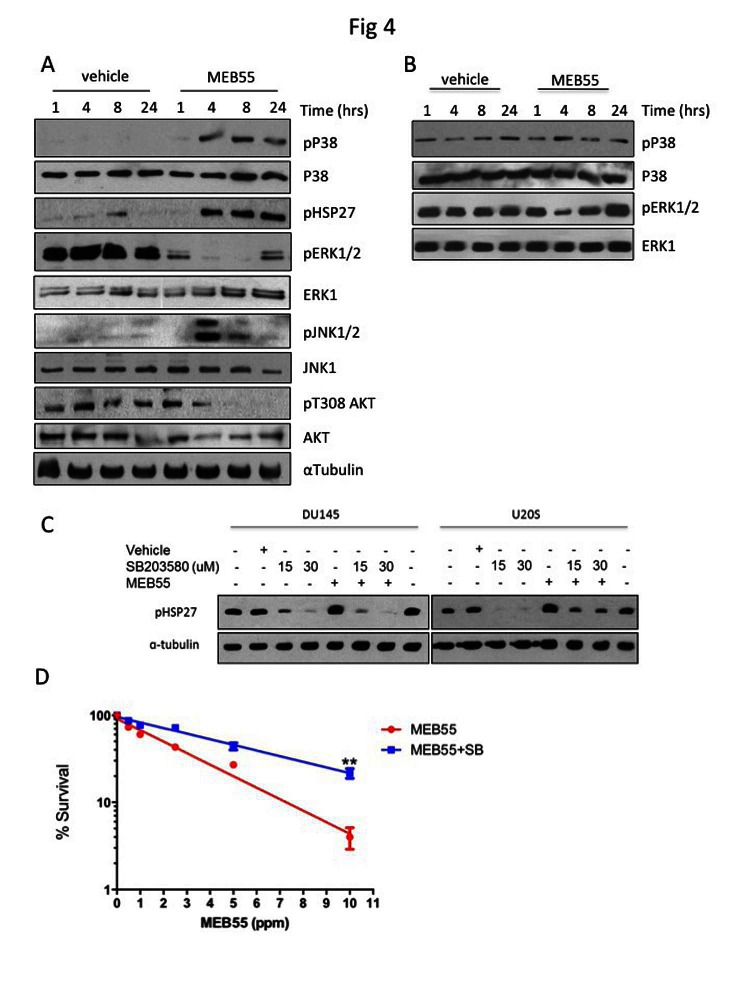
MEB55 modulates stress and survival signaling pathways. A. DU145 cells and B. BJ fibroblasts cells were treated with MEB55 (10 ppm) for the indicated times and resulting lysates were analyzed by immunoblot analysis with the indicated antibodies. C. DU145 and U2OS cells were pretreated for 1 hr with SB203580 at the indicated doses. After which time media was replaced with fresh media supplemented with SB203580 alone or in combination with MEB55 (DU145, 10 ppm; U2OS, 5 ppm). Cells were incubated for a further of 6 hrs and HSP27 phosphorylation was analyzed by immunoblotting. D. Colony survival of DU145 cells as measured after pre-treatment with 50 μM SB203580 for 2 hrs and then exposure to different concentrations of SL for additional 4 hrs. Cells were trypsinized, re-plated in triplicates and cultured for 14 days. SB203580 was replenished every other day. Colonies with >50 cells were counted. Results represent mean of nine replicates ±SD. **, P<0.01 as analyzed by linear regression and Student *t-*test.

Members of the mitogen-activated protein kinases (MAPK) superfamily members play a pivotal role in cell survival and cell death. While ERK1/2 kinases are mostly involved in cell survival, the stress-related MAPK kinases, JNK1/2 and p38 elicit either pro- or anti apoptotic signals depending on signal intensity and tissue type [11]. Following treatment of DU145 cells with MEB55 for 1, 4, 8 or 24 hrs, the expression and phosphorylation of the three main arms of the MAPK family (Erk1/2; JNK1/2 and p38) were analyzed by immunoblotting (Figure 4A, Figure S4). p38 MAPK phosphorylation (pp38) was first evident at 4 hrs of SL treatment and remained elevated at 24 hrs. Phosphorylation of HSP27, a downstream target of p38 [25], on Ser82 was increased in a similar time-dependent manner as pp38 (Figure 4A). JNK1/2 displayed an acute and robust (15 fold increase) phosphorylation at 4 hrs, which decreased by 50% at 8 hrs and returned to basal levels at 24 hrs. In contrast, pERK1/2 levels were reduced 4 fold after 1 hr of treatment and decreased further between 4 and 8 hrs. The levels of another survival factor, phosphorylated AKT/PKB, were decreased 6 fold at 8 hrs and further decreased to undetectable levels at 24 hrs (Figure S4). While similar pattern of signaling activation was obtained in HCT116 cells (data not shown), in the non-immortalized ‘normal’ BJ fibroblast line (Figure 4B) that are resistant to synthetic SL analogue [3], pp38 levels remained largely unchanged and pERK1/2 levels were decreased only at 4 hrs.

We next tested whether activation of p38 stress-activated MAPK and/or JNK1/2 were required for SL induced growth inhibition and apoptotic induction. DU145 and U2OS cells were pre-treated with pharmacological inhibitors of p38 (SB203580) and JNK1/2 (SP600125) prior to the addition of MEB55. While SB203580 was able to completely block pHSP27 phosphorylation (Figure 4C), SP600125, only partially reduced the activation of JNK1/2 kinase (data not shown). Colony survival assays were performed in which U2OS cells were treated with 50 μM SB203580 for 2 hrs prior to being treated with varying doses of MEB55 for additional 4 hrs and then re-seeded in a limiting dilution to form colonies over 14 days. Treatment with SB203580 resulted in an incomplete but significant rescue of the SL inhibition of colony formation (Figure 4D), suggesting that both the p38/HSP27 axis and additional mechanisms are involved.

### Strigolactone induced stress response transcriptional program

**Figure 5:**
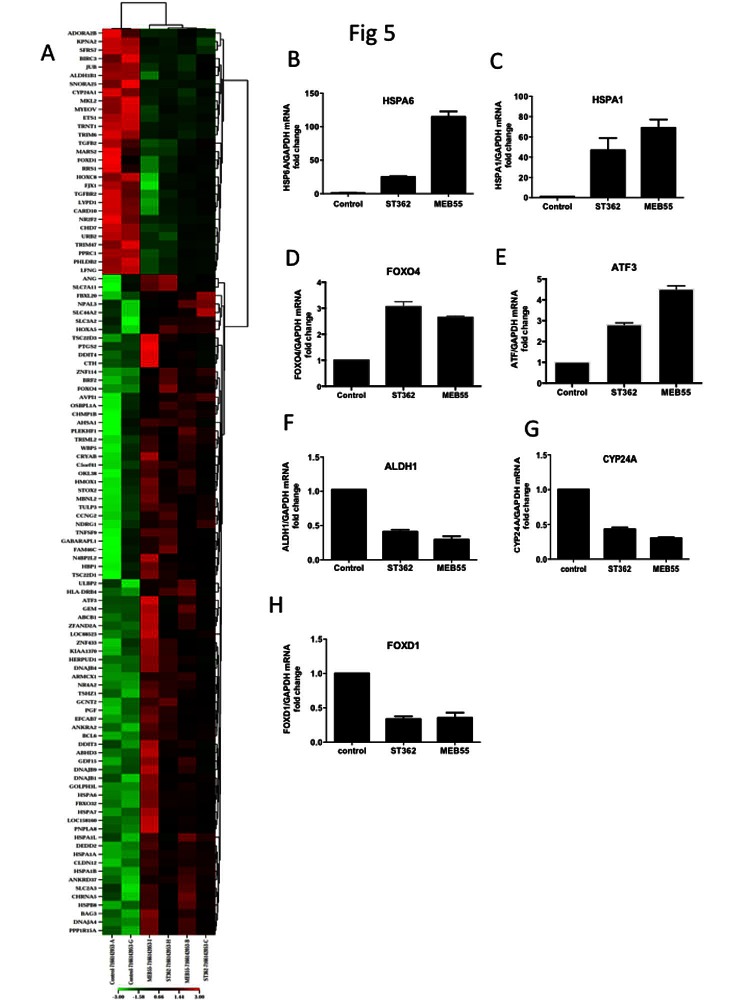

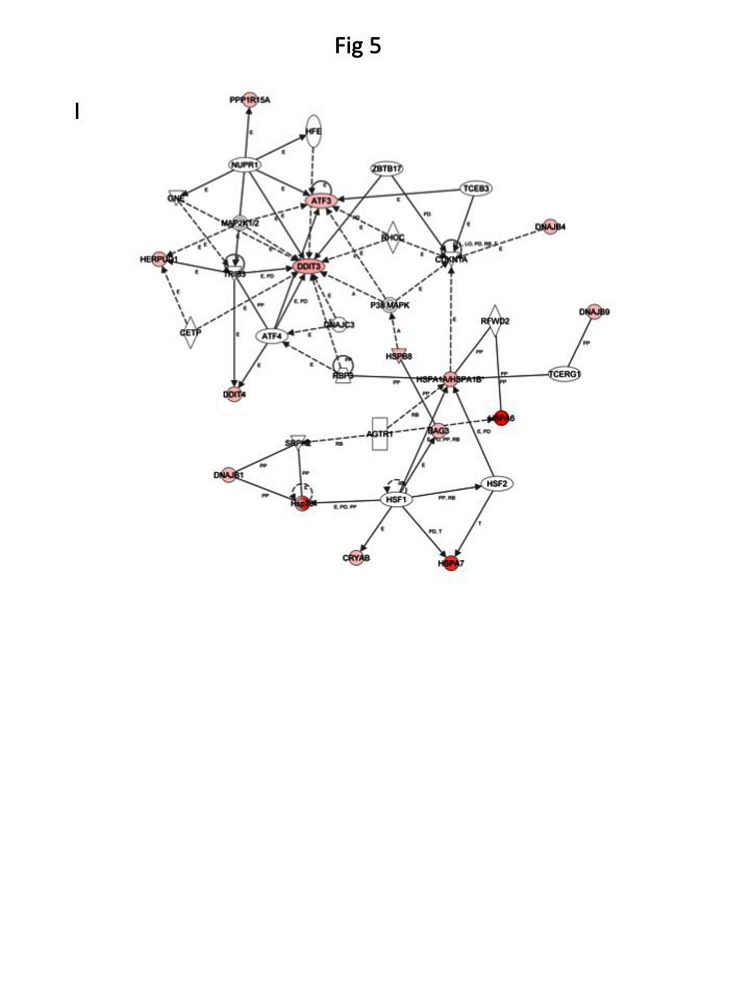
Global changes in gene expression following treatment with ST362 or MEB55. A. U2OS cells were treated with vehicle or 5 ppm of ST362 or 5 ppm MEB55 for 6 hrs. Total RNA was extracted and analyzed by expression array. Results of two independent experiments are displayed as a Heatmap of genes differentially expressed in U2OS cells treated with MEB55 or with ST362 for 6 hrs. Genes that were ±2 fold p≤0.05 upregulated are shown in red and down regulated transcripts are shown in green. A representative list of individual genes at 6 hrs (±2 fold p≤0.05) is presented in Table 2 and at 24 hrs in Table S1. B-H qRT-PCR analysis of representative genes in A. I. A protein network of top upregulated genes in response to MEB55 and ST362 was constructed using the Ingenuity software.

To assess the global transcriptomic effects of SL when growth inhibition and apoptosis of were initiated, U2OS cells were treated with 5 ppm of ST362 or MEB55 for either 6 (Figure 5) or 24 hrs (Supplementary Table 2) to permit early and late gene expression changes to be distinguished. A marked stress response was observed after 6 hrs of SL exposure with elevated expression of heat shock proteins (HSPA6, HSPA7, HSP1A, HSP1B, HSPB8, HSPA1L, AHSA1) (Table 2, Figure 5) and stress-related transcription factors (ATF3, FOXO1 and DDIT3). Changes in apoptosis regulating genes were also identified, including DDIT3, BIRC3 and BAG3. BIRC3 is an inhibitor of apoptosis and its expression was down regulated by both ST362 and MEB55. DDIT3 encodes a member of the CCAAT/enhancer-binding protein (C/EBP) family of transcription factors that is induced by stress including DNA damage. Moreover, DDIT3 over-expression can induce cell cycle arrest [32, 33] and indeed both MEB55 and ST362 treatment was associated with changes in cell cycle-related gene expression. MEB55 treatment was associated with increased expression of p21cip (CDKN1A), Cyclin F (CCNF), Cyclin A2 (CCNA2) and decreased expression of CDK6. Consistent with our earlier data, ST362 induced a modest down-regulation of Cyclin B1 (CCNB1) expression. Both MEB55 and ST362 induced Cyclin G2 (CCNG2), an unconventional cyclin homolog, which is linked to growth inhibition and whose expression is induced by DNA damaging chemotherapeutics [34]. SL exposure also induced changes in the expression of genes involved in metabolic functions in tumor and stem cells (ALDH1B1, ABCB1, SLC3A2, SLC44A2, SLC31A2, SLC7A11, CYP24A1, PTGS2/COX2, PPRC1), cytokines (CCL3L3, GDF15) and growth and differentiation factors (PGF, TGFBR11 and FOXD1).

At 24 hrs Supplementary Table 1, SL treatment was associated with down regulation of cell cycle regulators, including cyclins (CCNB2, CCNA2, CCNF) and cyclin regulatory proteins (CCNBP1 (cyclin B binding protein), CDKN3 (CDK inhibitor 3, a dual specificity phosphatase and an inhibitor of CDK2 activity), CDCA3, CDC20, CDC25C, CDCA2), the transcription factor E2F as well as mitotic genes involved in cytokinesis (KIF23, KIF4A, KIF11, KIFC1, KIF2C, KIF20A, IF15). The 24 hrs SL treatment resulted in up-regulation of genes involved in RNA processing and translation (RN7SK, SNORD3A, SNORD3C, SNORD3D) and altered expression of metabolic genes (DHRS2, SLC7A11, DUSP5, SCG5, ABCA13), growth factors (TGFB1, CTGF) and altered expression of genes involved in cellular adhesion (LAMA1, AMPH, ITGA2, SPP1/OPN1, ESM1, CYR61). ESM1 expression was the second (21.2-fold) and third (6.9-fold) most up-regulated gene in MEB55 and ST362 treated groups respectively. ESM1 is a secretory proteoglycan, whose expression is up-regulated by inflammatory cytokines. Altered expression of ESM1 has also been shown to induce cell cycle arrest [35] (Table S1). To validate the gene expression array data, qRT-PCR was carried out on 7 genes of which some were the most altered genes. HSPA6, HSPA1B, Cyp24A, ALDH1, ATF3, FOXD1 and FOXO4 exhibited similar expression pattern changes when analyzed by qRT-PCR as that observed by array analysis (Figure 5B-H).

Next we used the IPA database (Ingenuity® Systems, www.ingenuity.com) to identify networks and pathways of genes induced following 6 hrs exposure to SL analogues. The top up regulated genes confirmed a general up-regulation of stress and apoptotic responses that interestingly converge into the p38 pathway (Figure 5I).

### Strigolactone therapeutic potential

**Figure 6:**
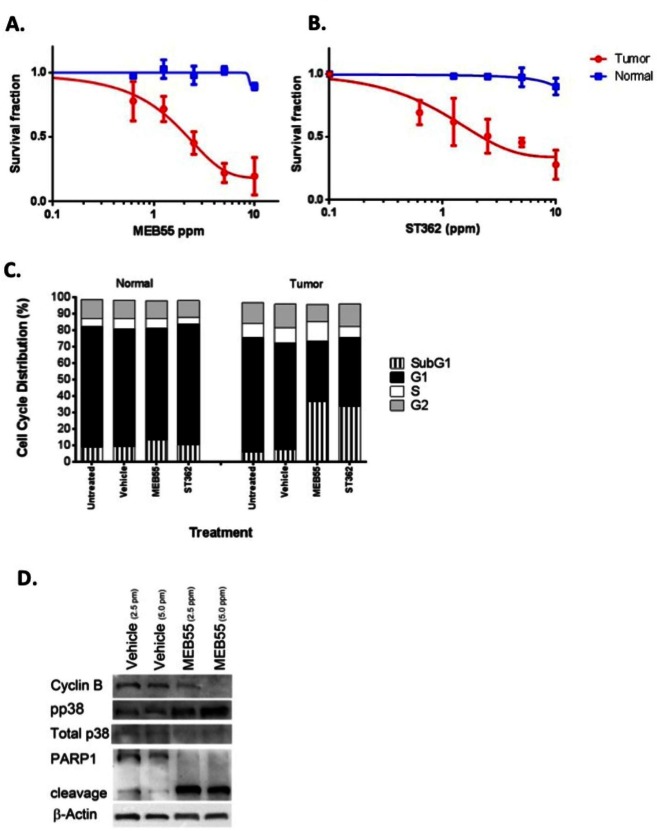
Enhanced sensitivity of primary prostate cancer cells to MEB55 and ST362. Normal prostate and prostate tumor CRCs were treated for 48 hrs with (A) MEB55 or (B) ST362. Cell viability was measured using the XTT cell viability assay. The IC_50_ of MEB55 is 1.8 ppm in tumor cells (95% confidence interval [CI], 0.294- to 0.427) and >20 ppm in normal cells (95% CI, 0.82 to 1.69), with statistical selectivity for tumor versus normal cells (P<0.0001)***. The IC_50_ for ST362 is 2.3 ppm in tumor CRCs (95% CI, 0.33-0.85) versus >20 ppm in normal CRCs (95% CI, 0.90 -1.1) with selectivity for tumor versus normal p<0.0001***. C. Cell cycle analysis of conditionally-reprogrammed normal and tumor prostate cells treated with IC_50_ concentrations of MEB55 or ST362 for 48 hrs. Cell Cycle analysis was performed by flow cytometry. D. Prostate tumor CRCs were treated with vehicle or the indicated concentrations of MEB55 were analyzed for changes in expression of cyclin B, total and phosphorylated p38 MAPK and PARP1 cleavage by immunoblot analysis.

To further explore the possible therapeutic potential of the SLs analogous, we utilized the novel model system of conditionally reprogramed cells (CRCs) [36], co-developed by us to permit the continuous culture of a wide spectrum of normal and malignant epithelium [28]. Both primary normal and tumor cells were obtained from the same patient and can be propagated indefinitely *in vitro* in the presence of irradiated murine 3T3 J2 fibroblast feeder cells, and Rho kinase inhibitor, Y-27632, as previously described [28, 29, 37]. Matched normal and tumor prostate cells were treated with different concentrations of SL analogues MEB55, ST362, ST357 and EG9 and the viability of cells was measured by XTT assays (Figure 6A, 6B and S5). All SLs reduced the viability of prostate tumor CRCs with MEB55 and ST362 being most potent and effective. The IC_50_ of MEB55 in the prostate tumor CRCs is 1.8 ppm 95% confidence interval [CI95%] 0.294-0.427 while the IC_50_ of MEB55 in normal prostate CRCs is extrapolated to be > 20 ppm and selectivity for tumor versus normal cells is highly significant at (p<0.001). Similarly, the IC_50_ of ST362 in tumor cells is 2.3 ppm [CI95%] 0.593 to 0.702 p< 0.001. The IC_50_ of ST357 in tumor cells is 5.649 ppm [CI95%] 0.647-0.826 p<0.001. None of the analogues caused more than 50% growth inhibition of normal prostate cells at the concentrations used.

**Table 2:** Classification of a representative list of SLs-responsive genes (6 hrs) with at least 2 fold change.

Function	ACCESSION	SYMBOL	DEFINITION	Fold-Change ST362 vs. control	p-value	Fold-Change MEB55 vs. control	p-value
Stress Response	NM_002155.3	HSPA6	Homo sapiens heat shock 70kDa protein 6 (HSP70B') (HSPA6), mRNA.	79.8	2.55E-05	158.7	1.09E-05
	NR_024151.1	HSPA7	Homo sapiens heat shock 70kDa protein 7 (HSP70B) (HSPA7), non-coding RNA.	23.3	0.000290977	55.7	7.45E-05
	NM_005346.3	HSPA1B	Homo sapiens heat shock 70kDa protein 1B (HSPA1B), mRNA.	9.2	2.21E-06	12.4	1.05E-06
	NM_005345.4	HSPA1A	Homo sapiens heat shock 70kDa protein 1A (HSPA1A), mRNA.	7.3	6.92E-07	12.4	1.05E-06
	NM_014365.2	HSPB8	Homo sapiens heat shock 22kDa protein 8 (HSPB8), mRNA.	2.7	1.43E-05	3.6	3.18E-06
	NM_005527.3	HSPA1L	Homo sapiens heat shock 70kDa protein 1-like (HSPA1L), mRNA.	2.7	0.00238603	3.9	0.00044493
	NM_012111.1	AHSA1	Homo sapiens AHA1, activator of heat shock 90kDa protein ATPase homolog 1 (yeast	2.1	0.0136702	2.6	0.0044504
Growth Factors	NM_002632.4	PGF	Homo sapiens placental growth factor (PGF), mRNA.	6.2	0.0001284	7.0	8.86E-05
Cytokines/ Signaling	NM_004864.1	GDF15	Homo sapiens growth differentiation factor 15 (GDF15), mRNA.	7.6	3.16E-05	19.0	3.66E-06
	NM_001001437.3	CCL3L3	Homo sapiens chemokine (C-C motif) ligand 3-like 3 (CCL3L3), mRNA.	3.7	0.0371505		
	NM_001024847.1	TGFBR2	Homo sapiens transforming growth factor, beta receptor II (70/80kDa) (TGFBR2), t	-2.0	0.0158406	-2.3	0.00859784
Apoptosis	NM_004083.4	DDIT3	Homo sapiens DNA-damage-inducible transcript 3 (DDIT3), mRNA.	6.2	0.000961245	14.1	0.000126234
	NM_004281.3	BAG3	Homo sapiens BCL2-associated athanogene 3 (BAG3), mRNA.	4.6	3.87E-05	7.7	7.20E-06
	NM_001165.3	BIRC3	Homo sapiens baculoviral IAP repeat-containing 3 (BIRC3), transcript variant 1,	-2.4	0.00864687	-3.2	0.002218
Cellular adhesion	NM_181702.1	GEM	Homo sapiens GTP binding protein overexpressed in skeletal muscle (GEM), transcript.	2.4	0.00147308	4.9	1.53E-05
	NM_012129.2	CLDN12	Homo sapiens claudin 12 (CLDN12), mRNA.	2.3	0.00116056	2.5	0.000758471
Cell Cycle Regulation	NM_004354.1 NM_031966.2	CCNG2 CCNB1	Homo sapiens cyclin G2 (CCNG2), mRNA. Homo sapiens cyclin B1 (CCNB1), mRNA	3.6 -2.1	1.59E-05 0.00562257	3.1	3.16E-05
Metabolism	NM_001013251.1	SLC3A2	Homo sapiens solute carrier family 3 (activators of dibasic and neutral amino ac	3.6	0.00297397	3.4	0.00378565
	NM_020428.2	SLC44A2	Homo sapiens solute carrier family 44, member 2 (SLC44A2), mRNA.	2.5	0.00318131	2.1	0.00883196
	NM_001860.2	SLC31A2	Homo sapiens solute carrier family 31 (copper transporters), member 2 (SLC31A2),	2.3	8.51E-05		
	NM_014331.3	SLC7A11	Homo sapiens solute carrier family 7, (cationic amino acid transporter, y+ syste	2.2	0.0388166	2.1	0.0461742
	NM_000927.3	ABCB1	Homo sapiens ATP-binding cassette, sub-family B (MDR/TAP), member 1 (ABCB1), mRN	3.6	0.000245971	8.1	1.49E-05
	NM_000963.1	PTGS2	Homo sapiens prostaglandin-endoperoxide synthase 2 (prostaglandin G/H synthase a	3.4	0.0294943	6.9	0.00419823
	NM_014330.2	PPP1R15A	Homo sapiens protein phosphatase 1, regulatory (inhibitor) subunit 15A (PPP1R15A	2.7	0.000380118	3.7	7.56E-05
	NM_000782.3	CYP24A1	Homo sapiens cytochrome P450, family 24, subfamily A, polypeptide 1 (CYP24A1), n	-2.6	0.000579055	-3.6	0.000113939
	NM_000692.3	ALDH1B1	Homo sapiens aldehyde dehydrogenase 1 family, member B1 (ALDH1B1), nuclear gene	-2.1	0.00210428	-3.1	0.00430455
Transcription	NM_001040619.1	ATF3	Homo sapiens activating transcription factor 3 (ATF3), transcript variant 4, mRN	2.2	0.0184042	4.0	0.00147967
	NM_005938.2	FOXO4	Homo s apiens forkhead box O4 (FOXO4), mRNA.	2.5	0.000437924	2.2	0.000948698
	NM_004472.2	FOXD1	Homo sapiens forkhead box D1 (FOXD1), mRNA.	-2.4	0.0361717	-2.7	0.0212245

Cell cycle analysis of primary normal and tumor prostate cells treated with vehicle, or with the identified IC_50_ concentration of MEB55 and ST362 indicated a significant increase in the subG1 fraction of tumor cells in response to MEB55 or ST362 (from 6% in control to 40% or 37% respectively, p<0.007), with only a slight increase in subG1 that was noted in normal cells (P =0.4) (Figure 6C). To identify the molecular changes associated with the cellular response of normal and tumor CRC cells to SLs, cells were treated with the IC_50_ concentrations of MEB55 followed by immunoblotting for cyclin B, pp38 as described above. Despite the lack of measureable G2/M cell cycle arrest, MEB55 caused a dramatic reduction in cyclin B expression in tumor CRC cells and a pronounced stress response was elicited by the three different SLs as determined by induction of pp38 (Figure 6D). In addition, the assay for PARP1 cleavage confirmed the robust apoptotic response observed in the tumor derived CRCs versus the patient-matched normal CRCs from the same patient (Figure 6D). Taken together, these data indicate that SL analogues can induce significant and non-reversible apoptotic response in both transformed cancer cell lines and in patient-derived tumor cells, while sparing normal cells and therefore may be useful therapeutic reagents.

## DISCUSSION

The present study sought to investigate the anti-tumorigenic effects of synthetic analogues of the strigolactone hormone towards human cancer cells. The array of cell lines used was chosen based on their diverse origin and oncogene expression status. We show that SL analogues inhibit the growth of different cancer cell lines including prostate, colon, lung and osteosarcomas. SLs also induced cellular stress response leading to cell cycle arrest and apoptosis in all tumor cells tested but not in normal fibroblasts. While the mechanisms of SLs growth inhibition only begin to unfold, our results indicate that SLs induce G2 cell cycle arrest in all cells regardless of their underlying genetic alterations, e.g. p53, k-ras or nuclear receptor status. We further show that SLs are effective in targeting human primary prostate cancer cells while being significantly less toxic to normal prostate cells of the same patient, suggesting that SLs might be treatment option in advanced prostate cancer.

SL-induced cell cycle arrest is likely mediated by down regulation of Cdc25C (Figure 2 and TableS1) and cyclin B1 mRNA and protein levels (Figure 2, Table 2 and Table S1). We found that down-regulation of cyclin B was partially rescued by proteasomal inhibition, suggesting that SLs regulate the expression of cyclin B at least in part by protein degradation via the ubiquitin-proteasome pathway. Interestingly, in Arabidopsis as well as in other plants, SLs inhibit root formation by down regulating cyclin B expression via the ubiquitin–proteasome pathway [38], suggesting the mechanisms of cyclin B regulation by SL are conserved between plants and mammals.

MEB55 and ST362 are the most potent SLs and induce apoptosis in all tested cancer cell lines. Significant loss of cell viability was initiated between 1 to 4 hrs of SLs exposure (depending on the cell line and analogue) and was non-reversible even after removal of the SLs. Notably, SLs induced apoptosis even at lower concentrations in the primary prostate tumor cells, which was confirmed by PARP1 cleavage. SLs had only minimal effect on the patient-matched primary normal prostate cells. We found that SLs induce the expression of several pro-apoptotic genes and inhibit the expression of several survival factors including the stemness marker, ALDH1, which is critical for viability and self-renewal of stem cells. These data support our previous finding that SLs inhibit self-renewal and are highly potent towards stem-like enriched cells [3]. We also noted the transient inhibition of ERK1/2 and a more durable inhibition of AKT in response to SLs. Inhibition of the PI3-K/AKT pathway has been shown to play an important role in the cytotoxic effects of another plant hormone, Methyl Jasmonate [39].

The activation of stress signaling is likely to be an important contributing factor to SLs’ induced cell cycle arrest and apoptosis. Array analysis revealed induction of multiple heat shock proteins and cytokines by 6 hrs following SLs exposure, which was validated by qRT-PCR. HSP6A and HSP7A, the highest inducible genes (20-158 fold induction), are members of HSP70 family that act to stabilize proteins against aggregation and misfolding. While many tumors express high levels of some HSP70 members, under extreme stress conditions and DNA damage, the induction of HSP expression, especially HSP6A contributes to cell cycle arrest and apoptosis [40]. Interestingly, a protein network constructed from the top up-regulated genes in response to SLs clearly indicate the activation of stress related network with p38 as the main focal point, further supporting our biochemical observations. In addition, the expression of stress-induced transcription factor, FOXO4, which is often phosphorylated and activated by JNK [41] is up regulated in response to SLs. Furthermore, we show that p38 MAPK and JNK1/2 were activated as early as 4 hrs following SL exposure. The functions of JNK and p38 MAPK in cancer cells are complex; P38 MAPK and JNK1/2 play a crucial role in cellular stress responses, signaling for cell cycle arrest [42] and apoptosis [17, 43], yet, in some cases p38 MAPK and JNK enzymes are associated with cell survival, aggressive cancer phenotypes including invasiveness. Interestingly, pharmacological inhibition of p38 MAPK only partially alleviated the inhibitory effects of SLs treatment, while pharmacological inhibition of JNK1/2 together with SLs was toxic. Taken together, these data indicate that additional pathways may be involved in mediating SLs growth inhibitory effects, and the precise mechanisms remain to be determined.

The innovative technology of conditionally reprogramed cells (CRCs) is an attractive system for drug discovery in the era of “targeted therapy” and “personalized medicine”.

This system of long term cultures of matched normal and cancer cells that originated from biopsies of the same patient was recently described [28, 29, 37] and enables evaluation of the therapeutic index of new compounds including toxicity and efficacy for individual patients.

Here we used matched cultures of normal and tumor prostate cells obtained from a patient diagnosed with gleasons grade 7 prostate cancer. We show enhanced sensitivity of tumor cells to the two most potent SLs: MEB55 and ST362 over normal cells, supporting the selectivity of these SLs seen in cancer cell lines. Taken together, our data suggest that SLs may be promising candidates as broad-spectrum anti-cancer agents eliciting cell cycle arrest and apoptosis via modulation of stress signaling and abrogation of cell survival pathways. Further studies are warranted to determine whether the current findings can be applicable to other cases of advanced prostate cancer.

## Methods

### Cell culture

Cells were grown at 37 °C in a humidified 5% CO_2_-95% air atmosphere. All tissue culture media and serum were purchased from Gibco (Invitrogen), unless otherwise indicated. LNCaP, DU145, PC3, A549 were maintained in RPMI supplemented with 10% FCS and HCT116, SW480, HT-29, U2OS (ATCC, Manassas) were maintained in DMEM supplemented with 10% FCS (Atlanta Biologicals).

### Conditionally reprogramed cells

Human radical prostatectomy samples were collected under the auspices and approval of the Massachusetts General Hospital Institutional Review Boards. Following detailed pathological analyses that documented that the tissue sections collected were nearly exclusively comprised of either tumor or normal cells, the specimens were processed and established using the CRC method as previously described [28, 29, 37]. The CRC cultures were maintained as described on irradiated 3T3 J2 murine fibroblasts in DMEM/F12-medium (Invitrogen) containing 10 mM Y-27632 (Enzo Life Sciences) and were passaged every 3–4 days due to rapid proliferation induced by the ROCK inhibitor. After removal of the feeder cells, confluent human prostate cells (HPCs) were detached by trypsin treatment and passaged 1:10 (4.2 × 10^5^ HPCs per 75-cm^2^ tissue culture flask) for 3 days or 1:20 (2.1 × 10^5^ HPCs per 75-cm^2^ flask) for 4 days onto freshly irradiated feeders.

### Strigolactone treatments and Reagents

Hormones were solubilized in Acetone (Sigma) at stock concentrations of 5000.0 ppm. Cells were treated at the indicated doses by diluting the hormone to the required highest concentration in the appropriate culture medium. Serial dilutions were performed for subsequent lower concentrations and final vehicle concentrations equalized. N-Acetyl-L-leucyl-L-leucyl-L-norleucinal (ALLN) was purchased from Sigma. SB203580 and SP600125 were purchased from Cell Signaling Technology (Danvers, MA). All inhibitors were solubilized in DMSO according to the manufacturer’s instructions.

### XTT Viability Assay

Cells were seeded into a 96 well plates at 1500 cells per well in triplicates in normal growing media. The following day media was replaced with phenol-free DMEM supplemented with 10% charcoal stripped serum and the indicated final concentrations of Strigolactone analogue or vehicle (acetone) alone. Cells were incubated for 3 days, at which time XTT (2, 3,-bis(2-methoxy-4-nitro-5-sulfophenyl)-5-[(phenylamino)-carbonyl]-2H-tetrazolium inner salt) reduction was used to quantify viability according to manufacturer’s instruction (ATCC). Cells were incubated with XTT reagent for 2-3 hrs at 37 °C in a humidified 5% CO_2_-95% air atmosphere. Absorbance was recorded by a photometer SPEKTRAFluor Plus, Tecan (Salzburg, Austria) at 450 nm with 750 nm of reference wavelength. Cell survival was estimated from the equation: % cell survival = 100 × (*A*t-*A*c), where *A*t and *A*c are the absorbencies (450nm) of the XTT colorimetric reaction (ATCC) in treated and control cultures respectively minus non-specific absorption measured at 750nm. Absorbance of medium alone was also deducted from specific readings.

### Cell Cycle Analysis and phosphoHistone-H3 staining

Cells were seeded at densities of 2 × 10^5^ cells (SW480, HT-29) or 5 x 10^5^ cells (HCT116) per well in 10% DMEM in 6-well plate culture dishes. The following day, the media was replaced with phenol-free DMEM supplemented with 10% charcoal-stripped FBS with the indicated concentrations of SL or vehicle alone (acetone). After 48 hrs, cells were washed twice with 1 x PBS (pH 7.4), centrifuged at 360g for 10 min at 4°C, and fixed in chilled ethanol (70%; v/v in PBS) with gentle vortex mixing. To determine their DNA contents, the cells were stained with 40 μg/ml propidium iodide (PI) and analyzed using a FACSCalibur flow cytometer and CellQuest analysis software (Becton Dickinson, San Jose, CA). Where phosphoHistone-H3 staining was carried out, cells were incubated with polyclonal antibody against phospho-Histone H3 and then with secondary Goat anti-rabbit IgG- conjugated to FITC prior to PI staining.

### Annexin V staining

Cells were cultured for 48 hrs under the same conditions as those used for the DNA content/cell cycle analysis. All the cells were collected and resuspended in 100 μL 1 X Annexin V Binding Buffer (BD Biosciences, San Jose, CA, USA). 2 μL FITC-Annexin V (BD Biosciences) was added and incubated for 10 mins in the dark (room temperature). Cells were then stained with PI (Sigma, Saint Louis, Missouri, USA) to a final concentration of 40 μg/mL and the cells were incubated at room temperature for 15 min in the dark. Then, 400 μL of Annexin V binding buffer were added and flow cytometry was performed using a BD FACSCalibur flow cytometer. Cells were considered to be apoptotic if they were Annexin V+/PI- (early apoptotic) and Annexin V+/PI+ (late apoptotic). Each analysis was performed using at least 20,000 events.

### Immunoblotting analysis

Whole cell lysates were prepared using a lysis buffer containing: 50 mM Tris-HCl (pH 7.5), 0.5% NP-40, 0.1% SDS, 0.25% sodium deoxycholate, 125 mM NaCl, 1 mM EDTA, 50 mM NaF, 1 mM sodium orthovanadate, 2.5 mM sodium pyrophosphate, 1 mM sodium β-glycerophosphate, 1 mM PMSF, and a protease inhibitor cocktail (Roche Molecular Biochemicals) and cleared by centrifugation. Protein concentration was determined using the BCA Protein Assay (Pierce), and 20–50 μg of lysate were separated in a 4–12% SDS-PAGE gel. After transfer, membranes were blocked for 30 min at room temperature in 5% BSA (Sigma) in Tris-buffered saline containing 0.1% Tween-20. Primary antibody was incubated for either 1.5 hrs at room temperature or overnight at 4°C. Secondary antibody was incubated for 30 min at room temperature, and proteins were visualized with West Pico Stable (ThermoScientific). All antibodies were used at 1:1000 dilution unless otherwise stated. pT308AKT, AKT, pT180/Y182 P38 MAPK, P38 MAPK, pERK1/2, ERK1. pS82HSP27, pT183/Y185 JNK1/2, JNK1, pT14 Cdk1, Cdk1, α-tubulin (1:20,000, Sigma-Aldrich), Cyclin B1 (Santa Cruz Biotechnologies) and horseradish peroxidase-conjugated anti-rabbit IgG and anti-mouse IgG (1:5,000, Jackson Immunoresearch). Densitometric quantifications of the immunoblots were carried out using ImageJ software (NIMH).

### Colony Survival Assay

U2OS cells were either pre-treated with 50 μM SB203580 for 2 hrs or treated with different doses of MEB55 for 6 hrs. Cells were then trypsinized and re-seeded in triplicates in limited dilutions of 2x10^3^ cells/well in 6 well plates. Cells were allowed to form colonies for 14 days during which SB203580 was replenished every other day. Cells were fixed and stained with crystal violet and 70% EtOH. Colonies of 50 cells or more were counted.

### Statistical Analysis

Results are presented as mean ± SD of replicate analyses and are either representative of, or inclusive of at least two independent experiments. Statistical comparisons between two experimental groups were performed using student’s t-test (2-tailed, paired) whilst multiple group comparisons were performed using analysis of variance (ANOVA). Data regarded as being significant when *P* < 0.05 (*). Higher power is significance (p<0.01**, p<0.001***) are also employed and indicated in each figure legend. IC_50_ doses for SLs were calculated by interpolation of the sigmoidal dose response curves (Graphpad Prism 6.0 software).

### Gene expression analysis

Total RNA was prepared from cells treated with either ST362 or MEB55 (5ppm) for either 6 hrs or 24 hrs using Trizol (Invitrogen, Carlsbad, CA, USA) according to the manufacturer’s protocol. RNA purity was assessed by an A_260_/A_280_ ratio of ≥1.9, and by the integrity of 18S and 28S rRNA using an Agilent microfluidic chip. Array analysis was carried out on cDNA prepared from equal amounts of RNA (5μg) prepared from duplicate sample sets. Labeled cRNA was denatured and fragmented at 94°C for 35min and then hybridized overnight to an Illumina human GeneChip representing approximately 14,000 annotated human genes. Expression microarray image was initially processed by Illuminia® GenomeStudio®, which outputs the pre-processed data. The pre-processed sample intensities then were imported into Partek® Genomics Suite™ for the differential analysis test. Two-way ANOVA model was utilized, and the two factors are Time (6-hrs, 24-hrs) and Drug Treatment (MEB55, ST362). Four contrasts of the “Drug Treatment – Time” interaction was tested, they are “MEB55-6hour vs. Control-6hour”, “MEB55-24 hrs vs. Control-24 hrs”, “ST362-6hour vs. Control-6hour”, “ST362-24hour vs. Control-24hour’. The combination of the raw p-value < 0.05 and absolute Fold change > 2 was set as the cutoff to select the significantly expressed genes in each of above contrasts. The Benjamini Hochberg multiple testing correction was utilized to calculate the False Discovery Rate (FDR) as a reference. Gene lists were refined by eliminating genes with signal intensities <100 (log2=6.64) in both comparison groups.

### Quantitative Real-Time Polymerase Chain Reaction (qRT-PCR)

Total RNA was extracted using Trizol (Invitrogen, Carlsbad, CA, USA) using the manufacturer’s protocol. 1μg RNA was reverse-transcribed in a total volume of 20 μl using the High Capacity cDNA kit (Invitrogen). Primers were designed using PrimerQuest software (Integrated DNA Technologies). PCR was performed in triplicate using an ABI-Prism 7900 instrument (Invitrogen, Foster City, CA) and SYBR Green (Invitrogen, Foster City, CA) according to the manufacturer’s protocol. The expression of each target gene was normalized to the expression of GAPDH mRNA and is presented as the ratio of the target gene to GAPDH RNA, expressed as 2-ΔCt, where Ct is the threshold cycle and ΔCt = Ct Target - Ct GAPDH. Experiments were repeated three times.

## SUPPLEMENTARY FIGURE AND TABLE



## References

[1] Gomez-Roldan V, Fermas S, Brewer PB, Puech-Pages V, Dun EA, Pillot JP, Letisse F, Matusova R, Danoun S, Portais JC, Bouwmeester H, Becard G, Beveridge CA, Rameau C, Rochange SF (2008). Strigolactone inhibition of shoot branching. Nature.

[2] Umehara M, Hanada A, Yoshida S, Akiyama K, Arite T, Takeda-Kamiya N, Magome H, Kamiya Y, Shirasu K, Yoneyama K, Kyozuka J, Yamaguchi S (2008). Inhibition of shoot branching by new terpenoid plant hormones. Nature.

[3] Pollock CB, Koltai H, Kapulnik Y, Prandi C, Yarden RI (2012). Strigolactones: a novel class of phytohormones that inhibit the growth and survival of breast cancer cells and breast cancer stem-like enriched mammosphere cells. Breast cancer research and treatment.

[4] Newman DJ, Cragg GM (2004). Advanced preclinical and clinical trials of natural products and related compounds from marine sources. Current medicinal chemistry.

[5] Skoog F, Strong FM, Miller CO (1965). Cytokinins. Science.

[6] Ishii Y, Sakai S, Honma Y (2003). Cytokinin-induced differentiation of human myeloid leukemia HL-60 cells is associated with the formation of nucleotides, but not with incorporation into DNA or RNA. Biochimica et biophysica acta.

[7] Cohen S, Flescher E (2009). Methyl jasmonate: a plant stress hormone as an anti-cancer drug. Phytochemistry.

[8] Goldin N, Arzoine L, Heyfets A, Israelson A, Zaslavsky Z, Bravman T, Bronner V, Notcovich A, Shoshan-Barmatz V, Flescher E (2008). Methyl jasmonate binds to and detaches mitochondria-bound hexokinase. Oncogene.

[9] Clouse SD, Sasse JM (1998). BRASSINOSTEROIDS: Essential Regulators of Plant Growth and Development. Annual review of plant physiology and plant molecular biology.

[10] Steigerova J, Oklestkova J, Levkova M, Rarova L, Kolar Z, Strnad M (2010). Brassinosteroids cause cell cycle arrest and apoptosis of human breast cancer cells. Chemico-biological interactions.

[11] Wagner EF, Nebreda AR (2009). Signal integration by JNK and p38 MAPK pathways in cancer development. Nature reviews Cancer.

[12] Chung KS, Han G, Kim BK, Kim HM, Yang JS, Ahn J, Lee K, Song KB, Won M (2013). A novel antitumor piperazine alkyl compound causes apoptosis by inducing RhoB expression via ROS-mediated c-Abl/p38 MAPK signaling. Cancer chemotherapy and pharmacology.

[13] Greenman C, Stephens P, Smith R, Dalgliesh GL, Hunter C, Bignell G, Davies H, Teague J, Butler A, Stevens C, Edkins S, O'Meara S, Vastrik I, Schmidt EE, Avis T, Barthorpe S (2007). Patterns of somatic mutation in human cancer genomes. Nature.

[14] Hui L, Bakiri L, Stepniak E, Wagner EF (2007). p38alpha: a suppressor of cell proliferation and tumorigenesis. Cell Cycle.

[15] Hui L, Bakiri L, Mairhorfer A, Schweifer N, Haslinger C, Kenner L, Komnenovic V, Scheuch H, Beug H, Wagner EF (2007). p38alpha suppresses normal and cancer cell proliferation by antagonizing the JNK-c-Jun pathway. Nature genetics.

[16] Ventura JJ, Tenbaum S, Perdiguero E, Huth M, Guerra C, Barbacid M, Pasparakis M, Nebreda AR (2007). p38 alpha MAP kinase is essential in lung stem and progenitor cell proliferation and differentiation. Nature genetics.

[17] Iyoda K, Sasaki Y, Horimoto M, Toyama T, Yakushijin T, Sakakibara M, Takehara T, Fujimoto J, Hori M, Wands JR, Hayashi N (2003). Involvement of the p38 mitogen-activated protein kinase cascade in hepatocellular carcinoma. Cancer.

[18] Sosa MS, Avivar-Valderas A, Bragado P, Wen HC, Aguirre-Ghiso JA (2011). ERK1/2 and p38alpha/beta signaling in tumor cell quiescence: opportunities to control dormant residual disease. Clinical cancer research : an official journal of the American Association for Cancer Research.

[19] Zhang Y, Guo Z, Du T, Chen J, Wang W, Xu K, Lin T, Huang H (2013). Prostate specific membrane antigen (PSMA): a novel modulator of p38 for proliferation, migration, and survival in prostate cancer cells. The Prostate.

[20] Milone MR, Pucci B, Bruzzese F, Carbone C, Piro G, Costantini S, Capone F, Leone A, Di Gennaro E, Caraglia M, Budillon A (2013). Acquired resistance to zoledronic acid and the parallel acquisition of an aggressive phenotype are mediated by p38-MAP kinase activation in prostate cancer cells. Cell Death Dis.

[21] Greenberg AK, Basu S, Hu J, Yie TA, Tchou-Wong KM, Rom WN, Lee TC (2002). Selective p38 activation in human non-small cell lung cancer. American journal of respiratory cell and molecular biology.

[22] Milone MR, Pucci B, Bruzzese F, Carbone C, Piro G, Costantini S, Capone F, Leone A, Di Gennaro E, Caraglia M, Budillon A (2013). Acquired resistance to zoledronic acid and the parallel acquisition of an aggressive phenotype are mediated by p38-MAP kinase activation in prostate cancer cells. Cell Death Dis.

[23] Konishi N, Shimada K, Nakamura M, Ishida E, Ota I, Tanaka N, Fujimoto K (2008). Function of JunB in transient amplifying cell senescence and progression of human prostate cancer. Clinical cancer research : an official journal of the American Association for Cancer Research.

[24] Vivanco I, Palaskas N, Tran C, Finn SP, Getz G, Kennedy NJ, Jiao J, Rose J, Xie W, Loda M, Golub T, Mellinghoff IK, Davis RJ, Wu H, Sawyers CL (2007). Identification of the JNK signaling pathway as a functional target of the tumor suppressor PTEN. Cancer cell.

[25] Garrido C, Gurbuxani S, Ravagnan L, Kroemer G (2001). Heat shock proteins: endogenous modulators of apoptotic cell death. Biochemical and biophysical research communications.

[26] Kim JH, Choi JS, Lee BH (2012). PI3K/Akt and MAPK pathways evoke activation of FoxO transcription factor to undergo neuronal apoptosis in brain of the silkworm Bombyx mori (Lepidoptera: Bombycidae). Cell Mol Biol (Noisy-le-grand).

[27] Solimini NL, Luo J, Elledge SJ (2007). Non-oncogene addiction and the stress phenotype of cancer cells. Cell.

[28] Liu X, Ory V, Chapman S, Yuan H, Albanese C, Kallakury B, Timofeeva OA, Nealon C, Dakic A, Simic V, Haddad BR, Rhim JS, Dritschilo A, Riegel A, McBride A, Schlegel R (2012). ROCK inhibitor and feeder cells induce the conditional reprogramming of epithelial cells. The American journal of pathology.

[29] Yuan H, Myers S, Wang J, Zhou D, Woo JA, Kallakury B, Ju A, Bazylewicz M, Carter YM, Albanese C, Grant N, Shad A, Dritschilo A, Liu X, Schlegel R (2012). Use of reprogrammed cells to identify therapy for respiratory papillomatosis. The New England journal of medicine.

[30] Wei Y, Mizzen CA, Cook RG, Gorovsky MA, Allis CD (1998). Phosphorylation of histone H3 at serine 10 is correlated with chromosome condensation during mitosis and meiosis in Tetrahymena. Proceedings of the National Academy of Sciences of the United States of America.

[31] Acquaviva C, Pines J (2006). The anaphase-promoting complex/cyclosome: APC/C. J Cell Sci.

[32] Barone MV, Crozat A, Tabaee A, Philipson L, Ron D (1994). CHOP (GADD153) and its oncogenic variant, TLS-CHOP, have opposing effects on the induction of G1/S arrest. Genes & development.

[33] Fornace AJ, Nebert DW, Hollander MC, Luethy JD, Papathanasiou M, Fargnoli J, Holbrook NJ (1989). Mammalian genes coordinately regulated by growth arrest signals and DNA-damaging agents. Molecular and cellular biology.

[34] Zimmermann M, Arachchige-Don AS, Donaldson MS, Dallapiazza RF, Cowan CE, Horne MC (2012). Elevated cyclin G2 expression intersects with DNA damage checkpoint signaling and is required for a potent G2/M checkpoint arrest response to doxorubicin. The Journal of biological chemistry.

[35] Kang YH, Ji NY, Lee CI, Lee HG, Kim JW, Yeom YI, Kim DG, Yoon SK, Kim JW, Park PJ, Song EY (2011). ESM-1 silencing decreased cell survival, migration, and invasion and modulated cell cycle progression in hepatocellular carcinoma. Amino acids.

[36] Vaziri H, Chapman KB, Guigova A, Teichroeb J, Lacher MD, Sternberg H, Singec I, Briggs L, Wheeler J, Sampathkumar J, Gonzalez R, Larocca D, Murai J, Snyder E, Andrews WH, Funk WD (2010). Spontaneous reversal of the developmental aging of normal human cells following transcriptional reprogramming. Regenerative medicine.

[37] Suprynowicz FA, Upadhyay G, Krawczyk E, Kramer SC, Hebert JD, Liu X, Yuan H, Cheluvaraju C, Clapp PW, Boucher RC, Kamonjoh CM, Randell SH, Schlegel R (2012). Conditionally reprogrammed cells represent a stem-like state of adult epithelial cells. Proceedings of the National Academy of Sciences of the United States of America.

[38] Rasmussen A, Mason MG, de Cuyper C, Brewer PB, Herold S, Agusti J, Geelen D, Greb T, Goormachtig S, Beeckman T, Beveridge CA (2012). Strigolactones suppress adventitious rooting in Arabidopsis and pea. Plant physiology.

[39] Elia U, Flescher E (2008). PI3K/Akt pathway activation attenuates the cytotoxic effect of methyl jasmonate toward sarcoma cells. Neoplasia.

[40] Murphy ME (2013). The HSP70 family and cancer. Carcinogenesis.

[41] Essers MA, Weijzen S, de Vries-Smits AM, Saarloos I, de Ruiter ND, Bos JL, Burgering BM (2004). FOXO transcription factor activation by oxidative stress mediated by the small GTPase Ral and JNK. The EMBO journal.

[42] Correze C, Blondeau JP, Pomerance M (2005). p38 mitogen-activated protein kinase contributes to cell cycle regulation by cAMP in FRTL-5 thyroid cells. European journal of endocrinology / European Federation of Endocrine Societies.

[43] Chang HL, Wu YC, Su JH, Yeh YT, Yuan SS (2008). Protoapigenone, a novel flavonoid, induces apoptosis in human prostate cancer cells through activation of p38 mitogen-activated protein kinase and c-Jun NH2-terminal kinase 1/2. The Journal of pharmacology and experimental therapeutics.

