# Supplementary material for: Strigolactone analogues induce apoptosis through activation of p38 and the stress response pathway in cancer cell lines and in conditionally reprogramed primary prostate cancer cells
Source: Oncotarget. 2014 Apr 2;5(6):1683–98. doi: 10.18632/oncotarget.1849 (PMC4039240; doi:10.18632/oncotarget.1849)
Supplement: Supplementary file 1 [file oncotarget-05-1683-s001.pdf]

Supplementary Figures

Figure 1:

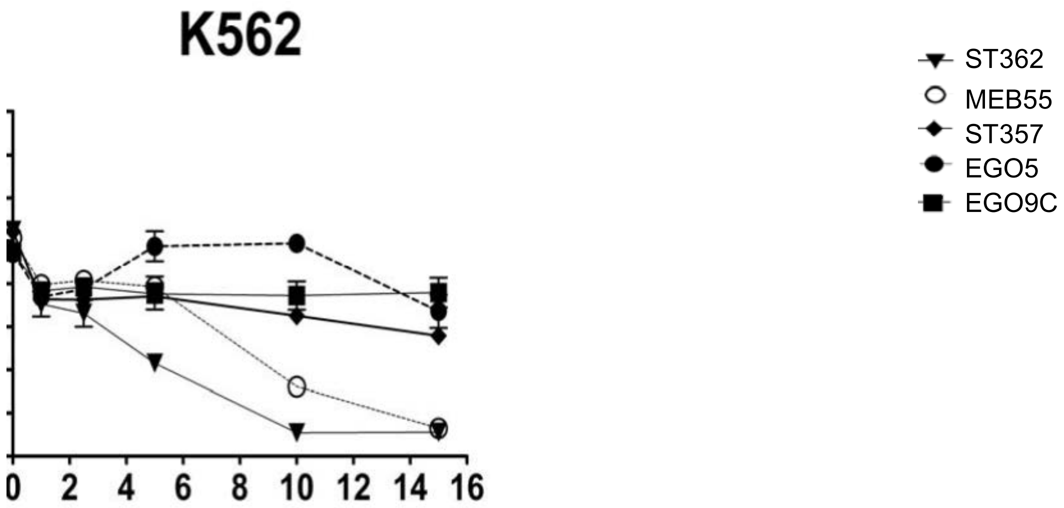

Figure S1: Effect of strigolactone analogues on human cancer cell line growth and viability. K562, Chronic Myeloid Leukemia cells were seeded into 96 well plates in normal growing media. On the following day, the media was replaced with phenol red-free DMEM supplemented with 10% charcoal stripped serum and the indicated doses of SL analogues or vehicle control alone were added. Cells viability was assayed after 3 days by XTT. Graphs are representative of at least two independent experiments with triplicate replicate wells for each analysis.

Figure 2

A

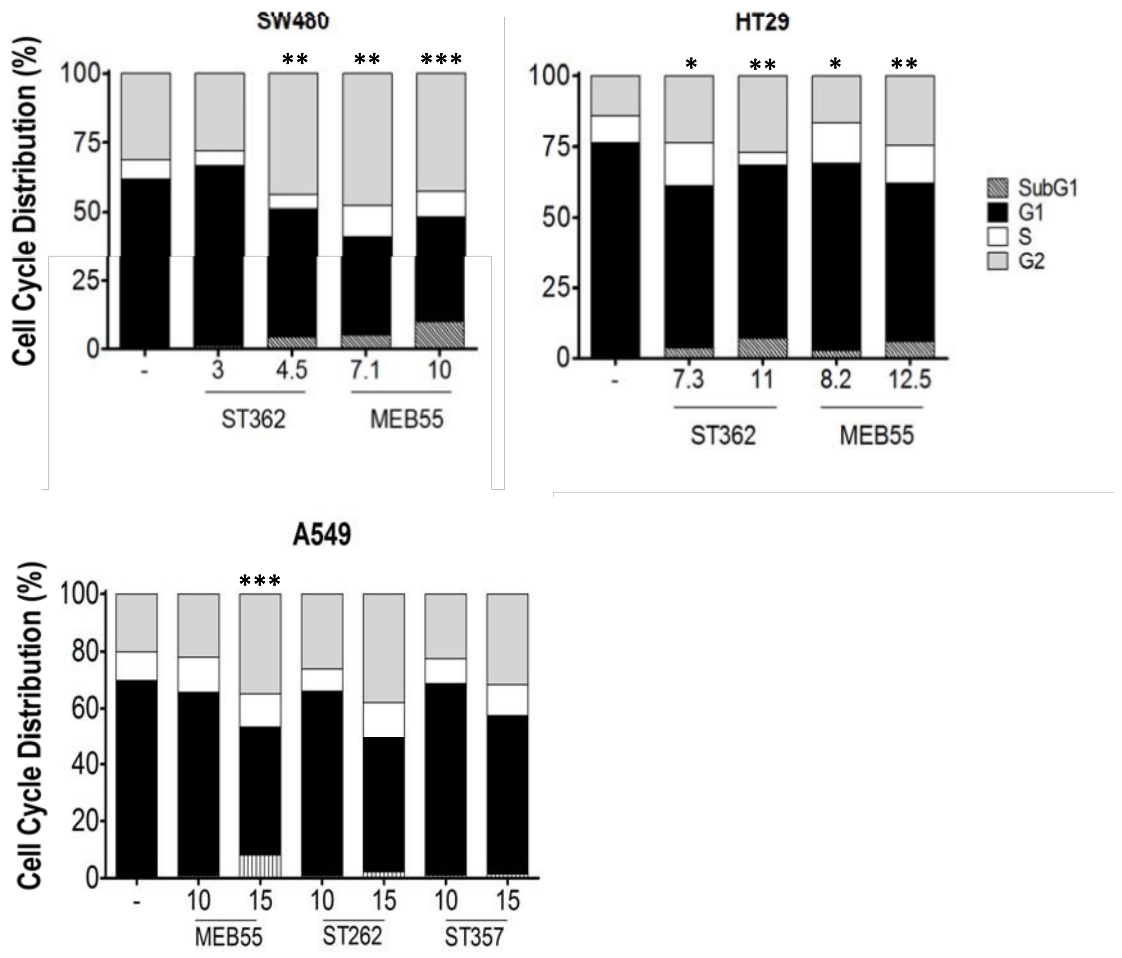

B

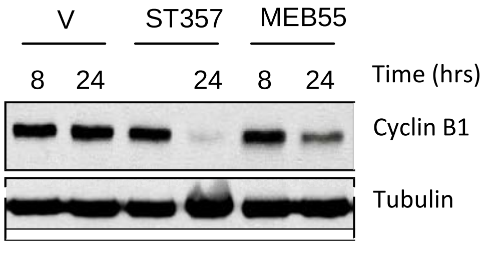

Figure S2: A. Cell cycle analysis of colon (HT29 , SW480) and lung cancer (A549) cell lines following SL treatment. Cells were seeded out at 3x10^5 cells per well into two 6-well plates in 10% DMEM. The following day media was replaced with DMEM supplemented with 10% charcoal stripped serum and the indicated doses of SL analogue. Cell were incubated for 48 hrs, fixed and then stained with propidium iodide. DNA content was analyzed by flow cytometry. Bar graphs represent mean of at least two experiments. *** P<0.001 ** p< 0.01 and * p<0.05 as analyzed by Student *t*-test. B. Cyclin B expression is down regulated by SL analogues. HCT116 cells were seeded into 6 well plates as described above and then treated with 10 ppm of SL analogues ST357 and MEB55 for the indicated times. Cell extracts were prepared and cyclin B levels were analyzed by western blot.

Figure 3:

C

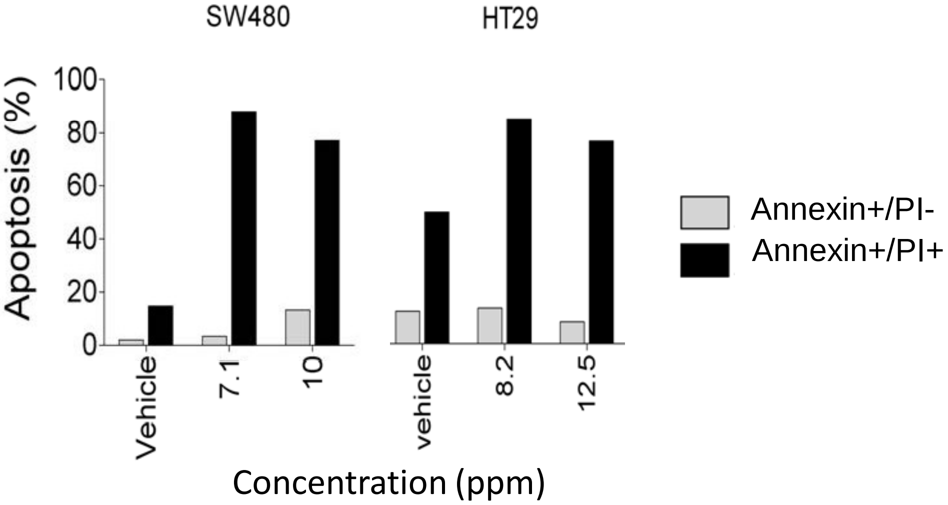

Figure S3: AnnexinV staining of SW480 and HT29 cells following 48 hour Strigolactones treatment. Cells were co-stained with annexin-V and propidium iodide and analyzed by flow cytometry. Bar graphs showing the distribution of cells in early (*gray bars*) and late apoptosis (*black bars*) following treatment with MEB55 at the indicated concentration (ppm).

Figure 4

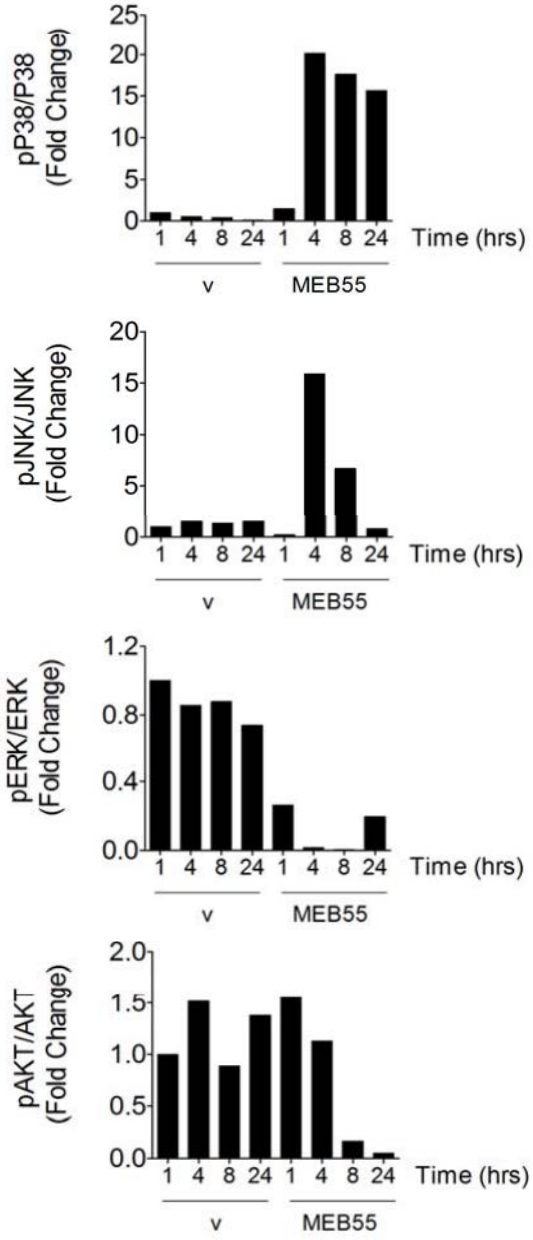

Figure S4: Analysis of DU145 cells following SL analogues treatment. Cells were treated with MEB55 (10ppm) for the indicated time points and resulting lysates were analyzed by immunoblot (Figure 5). Bars showing quantification of immunoblots (Figure 4) with ImageJ software.

Supplementary Table 1: Differentially Expressed Genes in Strigolactone treated cells at 24 hours

Function	SYMBOL	DEFINITION	Fold-Change ST362 vs.control	p-value	Fold-Change MEB55 vs. control	p-value
**Stress Response**	HSPA5	heat shock 70kDa protein 5 (glucose-regulated protein)	-2.8	0.0003223	-2.1	0.0020362
**Cytokines/ Signaling**	GDF15	growth differentiation factor 15	4.2	0.0002333	2.2	0.0046198
	CCL20	chemokine (C-C motif) ligand 20	6.6	3.63E-06	11.9	7.11E-07
**Metabolism**	SLC7A11	solute carrier family 7, (cationic amino acid transporter)	3.6	0.0053044	4.1	0.0031695
	DUSP5	dual specificity phosphatase 5	3.9	2.83E-05	3.8	3.26E-05
	SCG5	secretogranin V (7B2 protein)	3.0	0.0068693	4.9	0.001189
	DHRS2	dehydrogenase/reductase (SDR family) member 2	-2.5	0.0005996	-2.3	0.0009286
	ABCA13	ATP-binding cassette, sub-family A (ABC1), member 13	-2.8	0.0261932	-2.6	0.0340931
**Apoptosis**	BIRC3	baculoviral IAP repeat-containing 3	2.6	0.0062311	2.4	0.009596
**Growth Factors**	CTGF	connective tissue growth factor	2.4	0.0176348	2.1	0.0331052
	TGFBI	transforming growth factor, beta-induced	-3.4	0.0006391	-2.0	0.0091635
**Transcription**	E2F2	E2F transcription factor 2.	-3.3	0.0016412	-2.2	0.0108782
	EGR1	early growth response 1	2.2	0.0086051	2.7	0.0030771
**Cell Cycle**	KIF20A	kinesin family member 20A.	-4.7	5.56E-06	-2.4	0.0001626
	CCNB1	cyclin B1	-2.2	0.0044308		
	CCNG2	cyclin G2	2.7	6.45E-05		
**Cellular adhesion**	LAMA1	laminin, alpha 1	3.0	0.000149	2.1	0.0011095
	AMPH	amphiphysin	-3.5	0.0051844	-2.7	0.0143566
	ITGA2	integrin, alpha 2 (CD49B, alpha 2 subunit of VLA-2 receptor)	3.4	0.0018366	3.6	0.0013518
	SPP1	secreted phosphoprotein 1 (SPP1)	3.9	0.017545	6.6	0.0040549
	ESM1	endothelial cell-specific molecule 1	6.9	0.0007605	21.2	5.99E-05
	CYR61	cysteine-rich, angiogenic inducer, 61	3.6	0.0022875	2.2	0.0209306
**RNA Processing/ Translation**	RN7SK	RNA, 7SK small nuclear	16.8	3.50E-05	15.3	4.25E-05
	SNORD3A	small nucleolar RNA, C/D box 3A, small nucleolar RNA.	4.1	0.0002884	4.2	0.0002746
	SNORD3C	small nucleolar RNA, C/D box 3C small nucleolar RNA.	4.0	0.0002162	4.8	0.0001134
	SNORD3D	small nucleolar RNA, C/D box 3D small nucleolar RNA.	4.8	9.39E-05	4.8	9.39E-05

Figure 5

A.

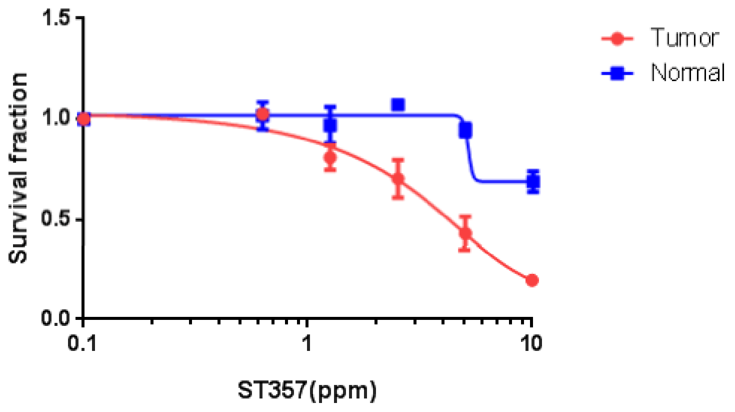

B.

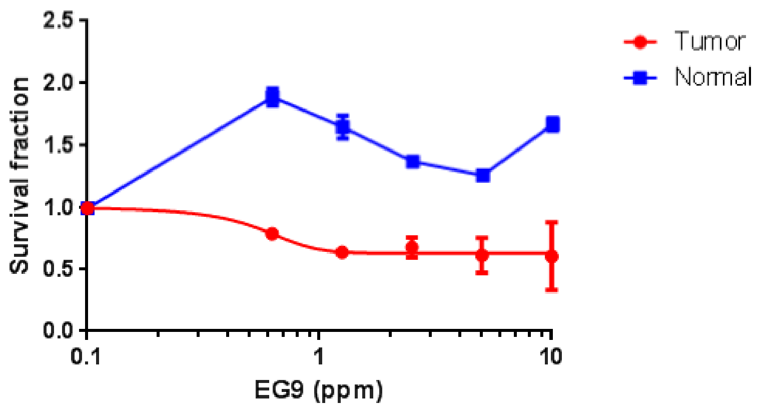

Figure S5: Sensitivity of prostate tumor CRCs to Strigolactone analogues.
Normal prostate CRCs and prostate tumor CRCs were treated with ST357 (A) or EG9c (B) at the indicated concentrations for 48 hrs. Cell viability was measured by XTT Cell Viability Assay. The IC_{50} of ST357 is 5.65 ppm in tumor cells (95% confidence interval [CI], 0.64- to 0.82) and >10 ppm in normal cells (95% CI, 0.97 to 1.06). The IC50 for EG9 is .10 ppm in tumor cells and >20 ppm in normal cells.
